# Lithium treatment for affective disorders: Exploring the potential of salivary therapeutic monitoring

**DOI:** 10.1016/j.nsa.2024.104067

**Published:** 2024-04-13

**Authors:** Maja P. Völker, Lea Sirignano, Helene Dukal, Alexander Schwesinger, Peter Findeisen, Fabian Schummer, Nils Hummel, Juliana Bresele, Michael Schneider, Joachim Behr, Thomas Stamm, Oliver Zolk, Anne Pietzner, Michael Hauptmann, Tino Graßhof, Michael Hartlep, Manfred Decker, Anne Müller, Frank Gerlach, Winfried Vonau, Stephanie H. Witt, Douglas A. Granger, Georgia M. Parkin, Elizabeth A. Thomas, Maria Gilles, Marcella Rietschel, Pichit Buspavanich

**Affiliations:** a Department of Genetic Epidemiology in Psychiatry, Central Institute of Mental Health, Medical Faculty Mannheim, Heidelberg University, Mannheim, Germany; b Department of Psychiatry and Psychotherapy, RG Stress, Central Institute of Mental Health, Medical Faculty Mannheim, University of Heidelberg, Germany; c MVZ Laboratory Limbach, Heidelberg, Germany; d Department of Psychiatry, Psychotherapy and Psychosomatics, University Hospital Ruppin-Brandenburg, Brandenburg Medical School Theodor Fontane, Faculty of Health Sciences Brandenburg, Neuruppin, Germany; e Department of Neurology, University Hospital Ruppin-Brandenburg, Brandenburg Medical School Theodor Fontane, Neuruppin, Germany; f University Clinic for Psychiatry and Psychotherapy, Brandenburg Medical School Theodor Fontane, Rüdersdorf, Germany; g Department of Psychiatry and Psychotherapy, Campus Charité Mitte, Research Department of Experimental and Molecular Psychiatry, Charité - Universitätsmedizin Berlin, Berlin, Germany; h Department of Psychology, Brandenburg Medical School Theodor Fontane, Faculty of Health Sciences Brandenburg, Neuruppin, Germany; i Department of Psychiatry and Psychotherapy, Campus Charité Mitte, Charité - Universitätsmedizin Berlin, Corporate Member of Freie Universitat Berlin and Humboldt-Universitat zu Berlin, Berlin, Germany; j Institute of Clinical Pharmacology, Brandenburg Medical School Theodor Fontane, Faculty of Health Sciences Brandenburg, Rüdersdorf, Germany; k Department of Medicine B, University Hospital Ruppin-Brandenburg, Brandenburg Medical School Theodor Fontane, Neuruppin, Germany; l Institute of Biostatistics and Registry Research, Brandenburg Medical School Theodor Fontane, Faculty of Health Sciences Brandenburg, Neuruppin, Germany; m Cubeoffice GmbH & Co.KG, Magdeburg, Germany; n Trace Analytics GmbH, Braunschweig, Germany; o Kurt-Schwabe-Institut für Mess- und Sensortechnik e.V. Meinsberg, Kurt-Schwabe-Straße 4, D- 04736, Waldheim, Germany; p Institute for Interdisciplinary Salivary Bioscience Research, University of California Irvine, Irvine, CA, USA; q Johns Hopkins University School of Nursing, Bloomberg School of Public Health, and School of Medicine, Baltimore, MD, USA; r Department of Neurosciences, University of California San Diego, San Diego, CA, USA; s Department of Epidemiology, University of California Irvine, Irvine, CA, USA; t Department of Psychiatry and Psychotherapy, Research Unit Gender in Medicine, Institute of Sexology and Sexual Medicine, Charité - Universitätsmedizin Berlin, Corporate Member of Freie Universitat Berlin and Humboldt-Universitat zu Berlin, Berlin, Germany

## Abstract

Lithium is one of the most effective medications for treatment-resistant depression and bipolar disorder. However, lithium has a narrow therapeutic window: overdosing can lead to life-threatening lithium intoxication while underdosing results in a lack of desired therapeutic effects. Therefore, lithium treatment requires close monitoring with regular laboratory assessments of blood serum concentrations. An alternative method for monitoring lithium at home would increase the safety and empowerment of patients. In our project, we are investigating the potential of saliva as a readily accessible medium. We are exploring a) the best method for collecting and processing saliva, b) methods to measure salivary lithium levels, and c) the correlation between blood serum and saliva concentrations of lithium, and influencing factors; with the aim to develop a Point-of-care home testing device.

## 1 Introduction

### 1.1 Lithium treatment in affective disorders

Lithium is an effective pharmacological treatment for major affective disorders: An extensive body of research has shown that lithium reduces suicide risk in patients with affective disorders (Lewitzka et al., 2015). For bipolar disorder (BPD), lithium is considered the gold standard therapy to prevent relapses into new episodes and treatment of acute manic episodes (Volkmann et al., 2020), while the evidence for the efficacy during acute episodes of depression, with mixed features, and rapid cycling is less consistent (Volkmann et al., 2020; Riedinger et al., 2023). In treatment-resistant depression, adding lithium to an antidepressant (lithium augmentation) has been found to be highly effective (Bauer et al., 2014; Nelson et al., 2014).

Even though guidelines recommend lithium for the treatment of treatment-resistant depression (Bauer et al., 2015), it is rarely prescribed as an augmentation agent (Nelson et al., 2014; Valenstein et. al, 2006). Although widely used for the treatment of bipolar disorder, lithium is underused in developing countries, and prescription rates are declining in developed countries (Shuy et al., 2024; Hidalgo-Mazzei et al., 2023). This could partially be explained by the narrow therapeutic window: while underdosing withholds treatment effects, an overdose can result in life-threatening lithium intoxication (Bauer et al., 2014). Moreover, lithium therapy frequently has negative effects on kidney function (Schoot et al., 2020), with an increasing risk of impaired kidney functioning for long-term lithium treatment and higher age (Osterland et al., 2023; Schoretsanitis et al., 2022). Additionally, lithium may have adverse effects on thyroid and parathyroid function (McKnight et al., 2012). Consequentially, patients treated with lithium are required to frequently visit a physician to monitor blood serum levels of lithium, and creatinine to calculate the glomerular filtration rate (eGFR) for kidney function (every 3–6 months), as well as serum levels of thyroid-stimulating hormone (TSH) for thyroid function, and calcium and parathyroid hormone (PTH) for parathyroid function (every 6–12 months) (Bauer et al., 2015; Schoot et al., 2020; McKnight et al., 2012). Especially in remote areas, monitoring of lithium treatment is highly inconvenient for both patient and physician. Additionally, although lithium is an affordable medication, the associated laboratory assessment is costly and time-consuming. This may also account for particularly low prescription rates in developing countries where resources for frequent monitoring might be lacking (Hidalgo-Mazzei et al., 2023). The dependence on clinical practices for therapeutic monitoring also limits patient empowerment. These factors partially account for rare prescriptions and high discontinuation rates in lithium therapy (Volkmann et al., 2020).To improve the feasibility of lithium treatment and make it more accessible for developing countries, an alternative method to determine lithium concentrations is needed.

Point-of-care (PoC) testing is the immediate testing of medical outcomes at the site of patient care (Vashist et al., 2015). In lithium treatment, PoC testing would eliminate the need for extensive laboratory analyses. This would enable faster decision-making, and increase safety for the patient. Moreover, allowing patients to self-monitor lithium levels at home would increase patient empowerment and doctor visits, especially if rapid and secure transmission of results to the treating physician is ensured. However, the feasibility of PoC testing at home has been limited by the requirement of trained personnel for blood serum collection. A more accessible biomaterial such as saliva would allow patients to monitor lithium and creatinine concentrations autonomously.

### 1.2 Previous attempts of lithium monitoring in saliva

Research interest in the potential of salivary monitoring of lithium treatment has started more than 30 years ago with several studies comparing lithium levels in saliva and blood serum (Kuczyńska et al., 2021). Saliva collection is non-invasive, cost-effective, and can be performed without a trained clinician.

In 2006, Serdarević and colleagues (Serdarević et al., 2006) collected saliva samples 2 and 12 h after the last lithium dose for comparison with blood serum in 25 patients. Salivary lithium concentrations were measured with both dry slide technology and atomic absorption spectrometry (AAS). Overall, salivary lithium levels exceeded serum levels by a factor of two to three. Higher correlations between saliva and serum levels were observed between the samples collected after 2 h (range = 0.936–0.938) compared to after 12 h (range = 0.822–0.860). Results from the dry slide technology and AAS were comparable.

In a study by Shetty and colleagues (Shetty et al., 2012), saliva was collected from 50 hospitalized patients with ongoing lithium therapy. Salivary lithium levels were analyzed with AAS. A correlation of 0.695 was found between salivary and serum concentrations of lithium. Higher correlations were shown in females compared to males.

Murru and colleagues (Murru et al., 2017) investigated the bioequivalence of blood serum and saliva in 38 bipolar patients with an ongoing lithium treatment of at least 4 weeks. Saliva flow was stimulated by chewing wax before collection. Lithium concentrations in the saliva were measured by AAS. Statistical analysis revealed a correlation of 0.767 between lithium concentrations in blood serum and saliva, with concentrations being higher in the saliva samples.

The most extensive study to date was published by Parkin and colleagues in 2021 (Parkin et al., 2021). At up to eight visits, saliva, blood serum, and demographic and medical data were collected from 75 subjects undergoing lithium treatment. Saliva concentrations were measured with Inductively Coupled Plasma Optical Emission Spectroscopy (ICP-OEP). They reported a correlation of 0.74 between salivary and serum concentrations. The correlations were affected by type 2 diabetes, daily lithium dose, and smoking. Moreover, they found that saliva concentrations predicted serum concentrations between (r = 0.70) and within patients (r = 0.71). Within-subject predictions improved significantly when using data on saliva-serum ratios of at least three preceding visits (r = 0.90).

Overall, the majority of studies suggest that saliva is a promising alternative to blood serum for monitoring lithium with the potential to be used in PoC testing at home. Nevertheless, caution is warranted due to inconsistent methodology, small sample sizes, and high variability in concentration ratios of saliva and blood serum (Kuczyńska et al., 2021; Parkin et al., 2021). The latter might be explained by a lack of control for demographic variables and medical history in most studies. Additionally, the mentioned studies do not address the need to monitor kidney function. To avoid frequent doctor visits, PoC testing in lithium treatment should also control for kidney function. As salivary sodium, and creatinine levels are associated with kidney impairment (Tomás et al., 2008; Bagalad et al., 2017; Anuradha et al., 2015), they are suitable analytes to measure renal function in salivary lithium monitoring and should be investigated along with salivary lithium levels. Overall, more longitudinal research, as well as collaborations among different researchers are necessary to establish reliability and validity, determine therapeutic concentration ranges of lithium, sodium, and creatinine in the saliva, and to determine the optimal protocols for saliva collection and analysis.

### 1.3 Aims

In the project consortium LiNaKre (Lithium-Natrium-Kreatinin; engl.: lithium-sodium-creatinine) funded by the German Federal Ministry of Education and Research (registration number: 13GW0484B), we aim to develop of a PoC device for monitoring lithium, sodium, and creatinine in saliva. The Kurt-Schwabe Institute for Measurement and Sensor Technology Meinsberg e.V. (KSI, Waldheim, Germany; https://www.ksi-meinsberg.de/) and Trace Analytics GmbH (Braunschweig, Germany; http://www.trace.de/) are currently developing a prototype for measuring relevant analytes in saliva. Additionally, cubeoffice GmbH & Co.KG (Magdeburg, Germany; https://cubeoffice.de/) are working on an application to document the patient's condition and lithium concentrations for fast transmission to the treating doctor. We (Brandenburg Medical School, Neuruppin, https://www.mhb-fontane.de/; Central Institute of Mental Health, Mannheim, Germany, https://www.zi-mannheim.de/) are responsible for clinical aspects of the study including specification of medical-diagnostic requirements.

For clinical aspects of the LiNaKre project, we intend to do additional explanatory research including genetic, epigenetic and, gene expression analyses as well as ambulatory assessment (AA) of mood and locomotion for a better characterization of depressive symptoms and remission. Following the suggestion of the ethical committee of the Brandenburg Medical School and the German Federal Institute for Drugs and Medical Devices (BfArM), a proposal for a reduced study design (without additional explanatory research) and a feasibility study conducted exclusively in Mannheim were submitted and approved (registration number Neuruppin: E−01-20210930; registration number Mannheim, 2022-565).

In the ongoing feasibility study, we are aiming to a) explore the best method for obtaining saliva in patients, b) establish and validate methods for measuring salivary lithium concentrations, and c) examine the correlation between lithium concentration in saliva and blood, and influencing factors.

## 2 Methods

### 2.1 Subjects and study design of the feasibility study

The study was approved by the ethical committee of the Medical Faculty Mannheim, University of Heidelberg. The planned sample size includes 20 patients. Inclusion criteria are a) 18–65 years of age, b) a diagnosis of unipolar depression or bipolar disorder, and c) ongoing or planned lithium therapy. Subjects with a) antisocial or other severe personality disorder, or schizophrenia, b) severe cognitive impairment, c) renal insufficiency stage 3 and higher (GFR 30–59), or severe cardiac or circulatory disease, or d) institutionalization by a court or official order are excluded.

### 2.2 Sample collection

In the feasibility study, sample collections take place during routine blood draws approximately once a week for the duration of hospital stay. Demographic data, psychiatric and somatic diagnoses, time of day of the collections, and medications are documented. First, 9 ml of blood serum is collected. With a pipette, blood is drawn from the needle tube and dropped onto two Whatman™ 903 Protein Saver Cards (Whatman plc, Maidstone, UK) to collect eight blood spots for future studies (Manfro et al., 2020). For saliva collection, the gold standard passive drool method is used (Granger et al., 2020). Subjects are instructed to avoid eating and drinking 30min before sample collection. They rinse the mouth with water 10min prior, then collect the saliva in the mouth and drool approximately 2.5 ml into a 5 ml Eppendorf tube (Eppendorf SE, Hamburg, Germany). The time for saliva collection should not exceed 2min. Additionally, patients fill out documentation of caffeine and food intake, smoking, sleep, and medication side effects.

### 2.3 Sample processing and analysis

All samples are processed after approximately 30min. The blood serum is centrifuged (2000×*g*; 15min, 18–25 °C), pipetted into seven aliquot tubes, and stored at −80 °C. The blood spots dry overnight and are stored at −20 °C. The saliva is vortexed for 10s and divided into two tubes. Following the protocol by Parkin and colleagues (Parkin et al., 2021) the first tube is frozen at −80 °C for at least 24 h, thawed, centrifuged (10,000×*g*; 4 °C, 5min), divided into four aliquots (min. 200 μl) and stored at −80 °C. The second half is filled in a syringe, pressed through a Rotilabo® syringe filter (PA55.1, pore size 0,20 μm, Ø 15 mm), and alliquoted into four tubes (200 μl each), and stored at −20 °C.

Lithium concentrations are analyzed using the Infinity™ Lithium liquid reagent on the AU680 Chemistry Analyzer (Beckman Coulter) with photometry. The obtained lithium concentrations will be compared to results of the routine blood testing.

## 3 Preliminary results

For the feasibility study, our first aim was to explore the best method for saliva processing. In a test trial with 10 patients at the Central Institute of Mental Health (CIMH, Mannheim, Germany), lithium and sodium concentrations in saliva were measured with photometry, and creatinine was measured with a kinetic colour test according to the Jaffé method at the Limbach laboratory in Heidelberg, Germany (https://www.labor-limbach.de/). The samples were frozen at −20 °C before transportation to the laboratory. While lithium, sodium and creatinine concentrations were detectable in the saliva, we found that the samples often lacked volume to measure lithium and were turbid, presumably due to a lack of pre-processing. Therefore, the standard operating protocol was adapted adding either a centrifugation step (10,000×*g*; 4 °C, 5min) and by a filtering step using Rotilabo® syringe filter; the latter being a method more feasible at home.

Since there is no established method of measuring lithium in saliva, we initiated a cooperation with leading salivary lithium researchers at the Institute for Interdisciplinary Salivary Bioscience Research, University of California Irvine, USA. As described above, they measured salivary lithium with ICP-OES in a prior study (Parkin et al., 2021). They re-analyzed saliva concentrations in a colorimetric assay (Abcam, ab235613) in a subset of n = 68 samples. We received a subset of n = 50 of their saliva samples to measure concentrations with the AU680 Chemistry Analyzer (Beckman Coulter) using the Infinity™ lithium reagent (Thermo Fisher Scientific Inc., Middletown, USA) at the Limbach laboratory, Heidelberg. As the Infinity™ lithium reagent so far only has been validated for blood serum, we wanted to validate this method for measuring lithium concentrations in saliva.

We found that saliva collection is easily built into clinical routine and is well-accepted by patients. However, providing the specified saliva volume for both processing methods with passive drool appears to be difficult for many patients. Therefore, a PoC device that requires small amounts of saliva for determining lithium concentrations would be desirable.

Secondly, we planned to establish and validate methods for measuring salivary lithium levels. The salivary lithium levels measured via ICP-OES by Parkin and colleagues (Parkin et al., 2021) showed a high agreement with levels measured in the colorimetric assay (r^2^ = 0.926), and with photometry at our site (r^2^ = 0.872). Our study validates the photometric method with the Infinity™ lithium reagent for measuring salivary lithium, which is already well-established for blood serum monitoring (for more details see Granger et al., 2023).

Regarding our last aim to test correlations between salivary and blood serum lithium concentrations as well as influencing factors, recruitment is ongoing and we have already collected several samples. After reaching our intended sample size of 20 patients, we will examine correlations and factors contributing to variability to replicate and validate the results by Parkin and colleagues (Parkin et al., 2021).

## 4 Outlook

After we have shown the feasibility of collecting saliva in the clinical routine and have established methods and protocols for sample processing, we are ready to start recruitment for the LiNaKre project at three different sites across Germany (Neuruppin, Rüdersdorf and Mannheim). Eventually, we plan to include 60 patients with up to 10 sample collections at different sites. The next step is to explore the relation between salivary and blood serum lithium concentrations and influencing factors.

Currently, factors that might influence salivary lithium concentrations are not investigated sufficiently. As AA, genetic, epigenetic, and gene expression analyses might inform about the influencing factors on lithium concentrations, we would like to revisit these factors in the future.

Moreover, no research has been performed so far on salivary lithium concentrations in patients with acute lithium intoxication. However, it is crucial to investigate whether lithium levels in saliva are linear in high concentration ranges. Therefore, we plan to recruit a cohort of patients with lithium intoxication to investigate how salivary concentrations change within extreme ranges.

In parallel, the development of a prototype for PoC testing by the KSI and Trace Analytics is proceeding within the LiNaKre project.

In addition to research on lithium concentrations in saliva, other studies have investigated the detectability of lithium in dried blood spots (DBS) (Manfro et al., 2020; Wikström et al., 2023). While Manfro and colleagues reported a modest correlation (r^2^ = 0.52) (Manfro et al., 2020), Wikström and colleagues even found a strong correlation between lithium concentrations in DBS and serum (r^2^ = 0.90) (Wikström et al., 2023). DBS might also provide a solution for annual or biannual monitoring of other markers such as sodium, potassium, calcium, TSH and PTH (Nicola et al., 2024). In the present study, blood spots were collected alongside to the saliva samples. The aim is to explore the potential of DBS beyond lithium monitoring in the future.

In conclusion, salivary lithium monitoring has great potential to improve treatment feasibility. However, high variations in serum-saliva ratios have been observed. Furthermore, the influence of factors such as additional medication, comorbid disorders and nutrition are not investigated sufficiently. The generation of more data is necessary for the implementation of a PoC device.

## Declaration of competing interest

The authors declare that they have no known competing financial interests or personal relationships that could have appeared to influence the work reported in this paper.
